# Research progress on the association between lymphoid hyperplasia and intussusception in the terminal ileum of children

**DOI:** 10.3389/fped.2026.1813286

**Published:** 2026-06-03

**Authors:** Dan Gao, Qingyu Xu, Nan Cong, Yi Song, Jia Li

**Affiliations:** 1 Department of Pathology, Hongqi Hospital Affiliated to Mudanjiang Medical University, Mudanjiang, Heilongjiang, China; 2 Department of Pediatric Surgery, Hongqi Hospital Affiliated to Mudanjiang Medical University, Mudanjiang, Heilongjiang, China; 3 Department of Pathology, Mudanjiang Tumor Hospital, Mudanjiang, Heilongjiang, China; 4 Graduate School of Mudanjiang Medical University, Mudanjiang, Heilongjiang, China; 5 Operating Room, Hongqi Hospital Affiliated to Mudanjiang Medical University, Mudanjiang, Heilongjiang, China

**Keywords:** children, ileocecal region, intussusception, lymphoid tissue hyperplasia, pathology

## Abstract

Lymphoid tissue hyperplasia in the ileocecal region of children is an important pathological factor associated with intussusception, and its mechanism involves multisystem interactions. The ileocecal region is a rich area of immune tissues in the infant intestinal tract. Under the influence of factors such as viral infection and inflammatory stimulation, lymphoid tissue can undergo reactive hyperplasia, resulting in changes in the morphology and biomechanical properties of the intestinal wall, which may become the initiating and promoting factors for intussusception. This article reviews the anatomical and physiological basis of ileocecal lymphoid tissue hyperplasia, its association with inducing factors such as infection, clinical manifestations, diagnostic methods, and treatment plans. In addition, frontier research directions are discussed, along with their potential value and challenges in clinical application. The aim of this article is to provide theoretical support and practical guidance for early clinical diagnosis and personalized precise treatment.

## 1 Introduction

Intussusception is one of the most common acute abdominal conditions in childhood and represents the leading cause of intestinal obstruction in children (1). Studies have shown that over 95% of cases are classified as primary intussusception (2), typically occurring in children aged 6 months to 2 years, with a male-to-female ratio of approximately 2–3:1 (3). Recent epidemiological studies have revealed seasonal variations (with higher incidence in spring and autumn) and regional differences, which may be related to viral prevalence and dietary habits (4). The ileocecal region is the area with the most abundant immune tissue in the intestine. Research has shown that the volume of lymphoid tissue in the ileocecal region of infants and young children may be significantly greater than that of adults, playing an important role in intestinal immune defense (5). In recent years, with advances in multimodal imaging techniques, molecular pathology, and computational biomechanics, the dual role of lymphoid tissue hyperplasia as both an initiating and –promoting factor in the pathogenesis of intussusception has received increasing attention. This article aims to provide a narrative summary of the relevant research progress, innovatively integrating the triad of immune microenvironment remodeling, abnormal mechanical force transmission, and intestinal microbiota dysbiosis as part of the pathogenesis model, thereby providing a new perspective for understanding intussusception. This article aims to provide evidence-based reference guidelines for clinical diagnosis and treatment.

## 2 Pathogenesis

### 2.1 Anatomical and physiological basis

The unique anatomical features of the ileocecal region in children involve not only its macroscopic structure but also its micro-biomechanical properties and the neuro-immune-endocrine regulatory network. In the ileocecal region, the intestine completes its rotation during the 10th–12th weeks of embryonic development. However, in infancy, the mesentery remains relatively long and incompletely fixed, resulting in significantly greater activity compared with adults. In addition, the ileocecal valve forms a lip-like protrusion of 1–1.5 cm within the cecum (6). Combined with the difference in diameter between the ileum and cecum during infancy, this makes the ileocecal valve more prominent within the cecum. When the intestine undergoes peristalsis, this relatively unstable anatomical structure, particularly the thickened ileocecal valve, is prone to being pushed forward, thereby causing entanglement of the intestinal tube. Moreover, studies have shown that the density of interstitial cells of Cajal in the ileocecal region may be lower than in other intestinal segments, which leads to slower conduction of slow-wave potentials. This increases susceptibility to coordination disorders of the peristaltic function and causing intestinal peristaltic rhythm disorders (7, 8). However, the specific contributions of these anatomical and electrophysiological differences in the occurrence of intussusception, as well as their synergistic interactions with lymphoid tissue hyperplasia, require further quantitative research.

### 2.2 Pathological basis of lymphoid tissue hyperplasia

Lymphoid tissue hyperplasia in the ileocecal region is an important pathological factor in pediatric intussusception. This region contains the richest immune tissue in the intestine, with a large number of Peyer's patches (PPs) and mesenteric lymph nodes. PPs consist of B-cell follicles, T-cell areas, and M cells, forming a complete immune unit (9). During infancy, lymphoid tissue in the ileocecal region naturally develops. When encountering viral infections (such as adenovirus or rotavirus), dietary changes, or inflammatory stimuli, the local lymphoid tissue will undergo reactive hyperplasia, resulting in thickening of the submucosa of the intestinal wall and hypertrophy of the ileocecal valve, thereby forming a “protrusion” in the intestinal lumen. These morphological changes not only directly reduce the diameter of the intestinal lumen but also disrupt the mechanical balance of the intestinal wall, leading to abnormal intestinal peristalsis rhythm. Ultimately, under the influence of intestinal peristalsis, the proximal intestinal segment is “pushed” into the distal intestinal lumen, thereby triggering intussusception (10). This process is not merely a simple morphological change. Hyperplastic lymphoid tissue, by altering the biomechanical properties of the local intestinal wall (such as elasticity and tension), may create a local “mechanical weakness,” which is more likely to become the starting point of intussusception during intestinal peristalsis. At present, there is a lack large-scale clinical imaging data on the specific volume threshold, spatial distribution pattern, and quantitative relationship between hyperplastic lymphoid tissue and intussusception risk.

### 2.3 Infection factors

Viral infection is a major factor contributing to the proliferation of lymphoid tissues in the ileocecal region (11). Studies have shown that acute intussusception is closely associated with infections caused by adenovirus, rotavirus, norovirus, and human bocavirus in the intestinal tract (12, 13). These viruses activate intestinal epithelial cells and dendritic cells through pattern recognition receptors (TLRs), thereby initiating the NF-κB and STAT3 signaling pathways, and leading to lymph node proliferation, edema, and congestion (14). The latest research has shown that levels of interferon-λ (IFN-λ) increase in the intestinal tract after viral infection. Although IFN-λ enhances antigen presentation, it also aggravates the inflammatory response (15). In addition, mesenteric lymphadenitis caused by bacteria such as *Salmonella* may spread throughout the body via lymphatic vessels, producing symptoms such as acute inflammation, bleeding, and necrosis (16). The relationship between lymphoid tissue proliferation and intussusception is not only influenced by viral or bacterial infections but also positively correlated with the type and quantity of the source of infection. It is worth noting that co-infection with enteroviruses and bacteria, such as adenovirus and pathogenic Escherichia coli, may increase the risk of intussusception and form a vicious cycle of “virus destroying the barrier–bacteria translocation–immune activation” (17).

### 2.4 Intestinal microbiota

An imbalance of the intestinal microbiota is regarded as an independent risk factor for intussusception. Existing studies have observed characteristic changes in the intestinal microbiota of children with intussusception, such as decreased α-diversity of the microbiota, decreased abundance of potentially beneficial bacteria (such as *Bifidobacterium* species), and increased relative abundance of potentially pro-inflammatory or opportunistic pathogenic bacteria (such as certain *Escherichia coli* strains, lipopolysaccharide-producing bacteria) (18, 19). Metabolomics studies have shown that concentrations of SCFAs in the feces of affected children are reduced, which may lead to a reduction in the expression of tight junction proteins in intestinal epithelial cells, thereby potentially increasing the permeability of the intestinal barrier and theoretically aggravating lymphoid tissue immune activation (20, 21). However, current studies on the association between microbiota and intussusception are mostly based on small-sample case–control or cross-sectional designs, and the research subjects are mainly children with the disease. These observational studies cannot completely exclude the confounding effects of the disease itself, treatment measures (such as fasting, antibiotic use), or dietary changes on the composition of the microbiota. There remains a lack of large prospective cohort studies from healthy children to clearly establish whether specific microbiota characteristics occur before intussusception and thereby establish a causal role. Therefore, it remains unclear whether microbiota imbalance is the cause or consequence of intussusception, and whether intervention of the microbiota can effectively prevent recurrence. Existing evidence is largely from observational studies, and prospective intervention trials are urgently needed to provide causal evidence. As shown in Table 1, adenovirus and rotavirus are most strongly associated with intussusception, suggesting that vaccination may have preventive value. While, microbiota imbalance as a potential risk factor, its specific mechanism still needs to be further clarified

**Table 1:** Major pathogens associated with lymphoid tissue hyperplasia in the ileocecal region and intussusception.

Type	Specific pathogens	Correlation strength and risk	Main pathogenic mechanisms	Reference
Virus	Adenovirus (Type 40/41)	Strong correlation, one of the most common pathogens	By activating the NF-κB/STAT3 pathway through TLRs, it causes congestion, edema, and hyperplasia of lymphoid tissues.	(22, 23)
Virus	Rotavirus	Strong correlation: The incidence rate decreases after vaccination	It damages the intestinal epithelial cells, activates the immune response, and causes intestinal peristalsis disorder.	(24, 25)
Virus	Norovirus	Moderate correlation	Similar to the mechanism of adenovirus, it triggers local inflammatory responses and lymphoid tissue hyperplasia.	(26)
Bacteria	Salmonella	Moderate correlation	It causes mesenteric lymph node inflammation, resulting in acute inflammation, bleeding, and necrosis. The pathogenic bacteria can be isolated.	(27)
Bacteria	Pathogenic *Escherichia coli*	The risk increases during co-infection	After the virus damages the intestinal barrier, bacterial translocation occurs, forming a vicious cycle of “virus–bacteria”.	(28)
Microecology	Microbial imbalance	Independent risk factor	The reduction of beneficial bacteria such as bifidobacteria leads to an imbalance in the intestinal microecology, which further aggravates immune activation.	(29)

### 2.5 Other inducing factors

Beyond infection factors, changes in diet are also an important trigger. During infancy, if complementary foods are introduced too early or in large amounts, due to the poor adaptability of the intestinal tract, it may lead to an excessive burden of antigens. The immune system of the intestinal tract relies on factors such as TGF-β and interleukins for tolerance toward food antigens. During infancy, this regulatory mechanism is not yet mature, making it prone to abnormal immune responses (30, 31). Studies have shown that early exposure to complex proteins, such as milk and egg proteins, may increase the risk of allergic reactions and stimulate the proliferation of mucosal-associated lymphoid tissues (32). In addition, drastic temperature fluctuations caused by climate change, accelerated intestinal transit due to diarrhea, increased cortisol levels due to stress suppressing the cholinergic anti-inflammatory pathway, and dysregulation of the autonomic nervous system are potential factors that can trigger intussusception.

The latest research indicates that the neuro-immune-endocrine axis plays an important role in physiological processes. In children with intussusception, the level of somatostatin in the plasma decreases, while gastrin significantly increases. This imbalance leads to an increase in the contraction frequency of intestinal smooth muscle and a decrease in contraction coordination, thereby causing gastrointestinal motility disorders and ultimately leading to the occurrence of intussusception (33). Moreover, serotonin, as an important neurotransmitter, plays a key role in immune regulation, inflammatory responses, and intestinal movement perception (34, 35). The excessive activation of 5-hydroxytryptamine neurons in the intestine not only promotes intestinal peristalsis but also inhibits the differentiation of Treg cells, forming a “neuro-immune positive feedback loop” (36).

## 3 Clinical manifestations

The typical clinical manifestations of intussusception caused by lymphoid tissue hyperplasia in the ileocecal region in children include paroxysmal crying, dark brown bloody stool, and a palpable mass in the abdomen, which constitute the typical “triad” of signs (37). Paroxysmal crying coincides with spasmodic contraction of the intestine, and the child can regain calm during the intervals between episodes. In the early stage of vomiting, it is mostly bile-like, suggesting proximal small intestinal obstruction. As the condition progresses, it may turn into foul-smelling vomiting, often indicating the possibility of intestinal ischemia (38). Dark brown bloody stool typically appears 6–12 h after onset, resulting from ischemic necrosis of the intestinal mucosa, bleeding, and mixing with intestinal secretions (39). Abdominal palpation often reveals a sausage-like, mildly tender mass in the right upper abdomen, and sometimes an empty feeling in the right lower abdomen (40). If the condition is severe, the mass may extend to the left lower abdomen and even prolapse through the anus (41).

Some children, particularly young infants, may present with atypical clinical manifestations, such as refusal to eat, drowsiness, and pale complexion without obvious crying or abdominal distension (42, 43). Rectal examination may reveal bloody stool, which is suggestive of intussusception. Newborns may present with jaundice and feeding difficulties, while children of school age may have atypical locations of abdominal pain. Some studies have explored D-lactic acid and intestinal-type fatty acid binding protein as potential biomarkers reflecting intestinal barrier damage, but their diagnostic value requires more clinical verification (44). A few cases may be related to superantigen reactions after viral infection, presenting with symptoms such as fever and rash (45). Repeated episodes of intussusception may lead to intestinal dysfunction and nutritional absorption disorders.

## 4 Diagnostic methods

### 4.1 Imaging examinations

**Figure 1:**
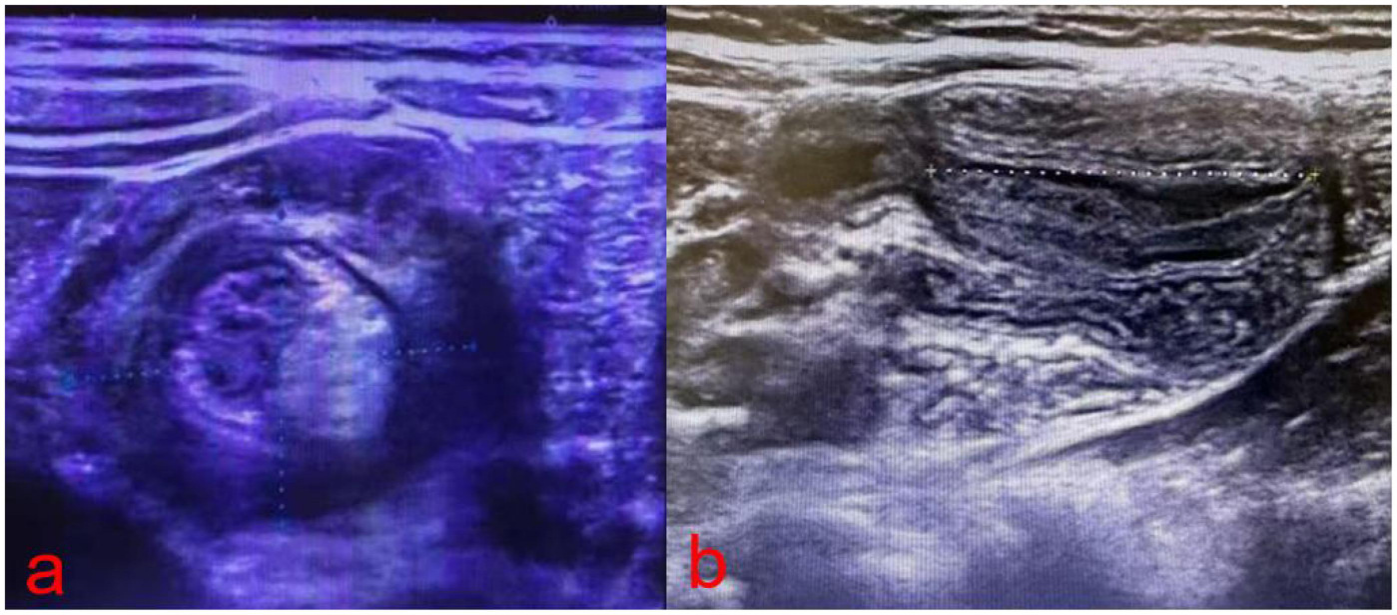
Cross-sectional ultrasound examination shows the “concentric circle sign” **(a)**, and the longitudinal section presents the “socket sign” **(b)**.

Abdominal ultrasound is the preferred imaging method for diagnosing intussusception. Its typical signs include the “concentric circle sign” on cross-sectional view and the “tube sign” on longitudinal sections, with a diagnostic sensitivity of up to 96% and specificity of 92% (46). As shown in Figure 1, these typical signs are not only direct evidence for diagnosis but also, through their clarity and completeness (such as the number of layers of the “concentric circle” and whether it is regular), indirectly reflect the degree of congestion and edema in the intussuscepted intestinal segment, providing preliminary imaging clues for evaluating intestinal vitality. High-frequency ultrasound can more clearly display the layered structure of the intestinal wall and submucosal edema (47). Yan et al. (48) demonstrated that shear wave elastography can quantitatively assess intestinal wall hardness, and correlation studies of its relationship with the length of intussusception suggests its potential as an auxiliary indicator for predicting the effect of enema repositioning.

Computed tomography (CT) is primarily used for complex cases or when ultrasound diagnosis is unclear. CT can accurately determine the location and length of intussusception, and whether there are complications, such as intestinal ischemia or perforation (49, 50). Through multi-planar reconstruction technology, the characteristics and specific location of intussusception can be clearly seen (51). By using dual-energy CT to quantitatively analyze the iodine concentration (IC) and the slope of the energy spectrum curve (λHU), the intestinal ischemia condition can be evaluated. Its sensitivity and specificity are superior to single-energy reconstruction (MR) (52, 53). Magnetic resonance imaging (MRI) is not considered a first-line examination method due to longer examination times and frequent need for sedation. However, its excellent soft-tissue resolution is valuable in the differential diagnosis of specific complex cases (54). Positron emission tomography–computed tomography is mainly used for the differential diagnosis of intussusception suspected to be secondary to malignant tumors (such as lymphoma) (55, 56). Artificial intelligence, particularly deep learning-based ultrasound image analysis systems, shows promise in assisting with the identification of intussusception characteristics and helps improve diagnostic efficiency, especially in primary medical settings (57).

### 4.2 Laboratory tests

Laboratory tests are primarily used to assist in evaluating a condition. Blood routine tests can provide information related to infection. If the white blood cell count and the proportion of neutrophils increase, it may indicate a bacterial infection (58). Through routine stool tests, red blood cells and white blood cells can be detected, which can assist in differential diagnosis (59). Fecal metagenomic sequencing technology can simultaneously identify viruses, bacteria, fungi, and drug resistance genes, with superior detection rates compared with traditional detection methods (60). The detection of C-reactive protein and procalcitonin helps determine the severity of the infection. Citrulline in the serum, as a marker of intestinal epithelial cell function, may indicate extensive mucosal damage when its concentration is lower than 19.07 μmol/L (61). In addition, the study of plasma exosomal miRNAs (such as miR-146a, miR-155) as liquid biopsy markers is still in the exploration stage (62).

### 4.3 Pathological examination

For children with recurrent episodes of intussusception (≥3 times) or suspected secondary causes, ultrasound endoscopy (EUS)-guided fine-needle aspiration biopsy can be considered to obtain histological evidence (63). The focus of pathological examination is to clearly identify the nature of lymphoid tissue hyperplasia, distinguishing between reactive hyperplasia and malignant lesions (64). In addition, immunohistochemical detection can assess the proliferative activity of lymphoid tissue, while *in situ* hybridization and PCR detection can help rule out lymphoma and further confirm the benign or malignant nature of the lesion. The results of genomic sequencing show that there are significant differences in biological behavior and gene expression profile between reactive hyperplasia of lymph nodes and malignant lymphoma, with the gene expression of malignant tumors being more unique (65).

**Figure 2:**
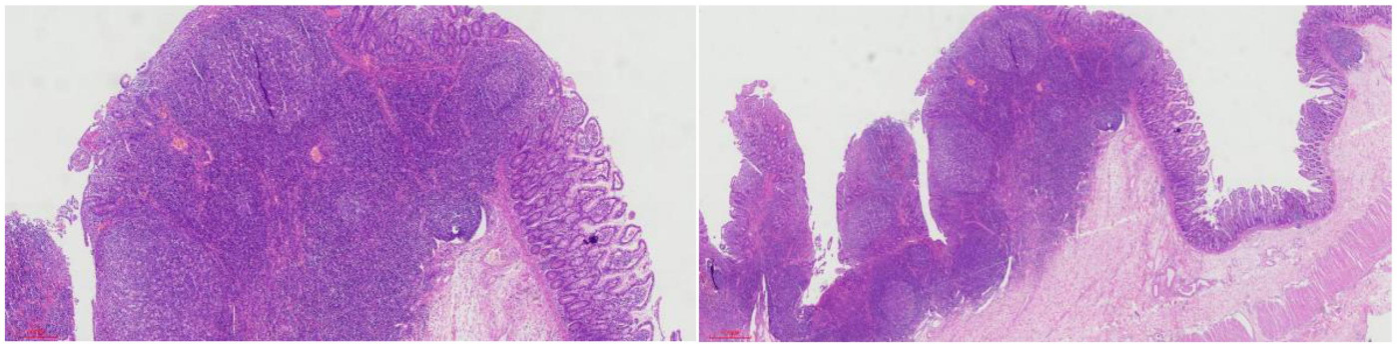
Pathological examination of the ileocecal region shows chronic inflammation of the terminal ileum mucosa, active lymphoid tissue hyperplasia in the Peyer's patch, and highly reactive hyperplasia of the lymph nodes.

Sun et al. (66) reported that secondary intussusception caused by malignant diseases typically has a longer course, higher incidence, and lesion located closer to the ileocecal region. The rapid frozen section technique used during surgery can assess the vitality of the intestinal tube, thereby providing further guidance for the extent of surgical resection. Near-infrared fluorescence imaging technology uses indocyanine green to assess intestinal perfusion and determine the boundary of intestinal necrosis based on baseline fluorescence intensity, thereby achieving precise resection (67). The most reliable diagnostic method remains detailed pathological examination of the lesion site after resection (as shown in Figure 2), which requires assessment of vascular and nerve invasion as well as the status of the resection margin, in order to provide molecular pathological evidence for subsequent treatment.

## 5 Treatment strategy

### 4.1 Non-surgical treatment

**Figure 3:**
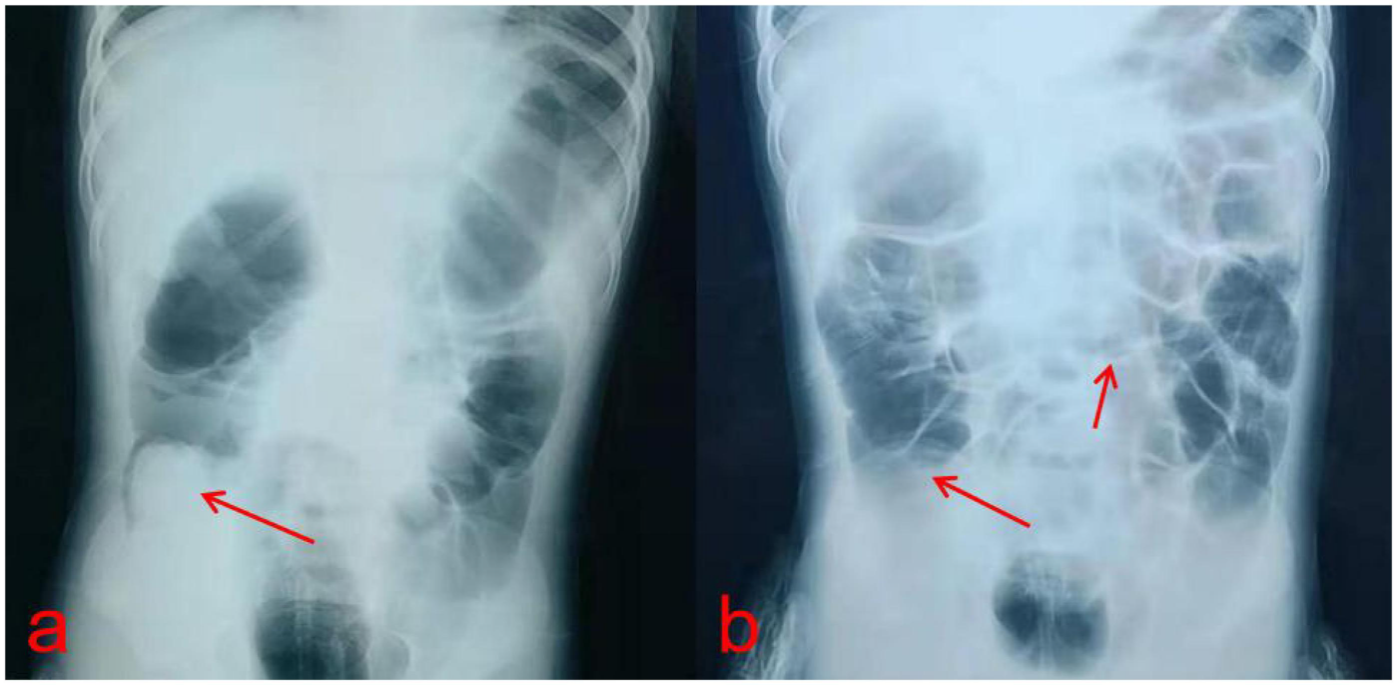
Comparison before and after air enema repositioning of intussusception. **(a)** Before repositioning, it demonstrated a “cup mouth sign”; **(b)** after repositioning, the sign disappeared and air entered the small intestine.

Air enema reduction is the preferred treatment for acute intussusception. In early cases, the success rate of air enema reduction can exceed 95% (68). However, when the disease duration exceeds 24 h or intestinal ischemia is present, the success rate will decrease significantly. When using automatic pressure control or manual timing colon inflation equipment, the initial pressure should be set between 6 and 8 kPa, with the maximum not exceeding 12 kPa to prevent intestinal perforation (69). A 16–20 F Foley catheter is inserted through the anus (70), an appropriate amount of gas is injected into the end balloon to fix the urinary catheter, and it is connected to the inflation equipment or pressure gauge. After gas injection, the soft tissue mass at the intussusception site will protrude semi-circularly into the colon, and a distinct cup-shaped shadow will form at the front end of the gas. As the pressure increases, the intussusception mass will gradually retract toward the cecum until it disappears completely (69). As shown in Figure 3, this schematic diagram not only visually demonstrates the dynamic retraction process of the intussusception head during the reduction process but also helps clinicians understand the importance of pressure control. Insufficient pressure may lead to failure of reduction, and excessive pressure, as mentioned previously, increases the risk of intestinal perforation. Therefore, Figure 3 offers a visual supplement to the safe operation procedure. Yan et al. (71) pointed out that in children with stable clinical conditions and no signs of intestinal perforation or shock, air enema is the preferred treatment. However, the success rate may be affected by factors such as the duration of symptoms, the type of intussusception, and the location of the intussusception (72). Water pressure enema under B-ultrasound monitoring, using 0.9% normal saline, with a pressure gradient controlled at 80–120 cmH_2_O, offers advantages of being mild, non-radiating, and repeatable, making it particularly suitable for infants (73, 74).

Retrograde intussusception reduction surgery under endoscopy is applicable for small intestinal intussusception. This procedure can be carried out under the visual assistance of small intestinal capsule endoscopy or magnetic navigation capsule endoscopy, and the intestinal entrapment can be gradually restored by the action of physiological saline or carbon dioxide pressure (75). In addition, in children with infections, targeted antiviral treatment, such as using cidofovir against adenovirus, can reduce lymphoid tissue edema and lower the risk of recurrence (76). Small-scale retrospective studies have shown that short-term use of glucocorticoids after intussusception reduction may help reduce the recurrence rate (77), but this still needs to be verified through larger-scale randomized controlled trials.

### 5.2 Surgical treatment

Surgical intervention is indicated in children for whom non-surgical treatment fails or who present with specific high-risk indications. The main indications include the following: (1) failure of air or water pressure enema reduction (78); (2) onset time exceeding 24–48 h, with clinical suspicion of intestinal necrosis or perforation (79); (3) recurrent (≥3 times) intussusception (78); (4) small intestinal intussusception (80); and (5) presence of organic lesions (such as Meckel's diverticulum, intestinal duplication, polyps, or tumors) (81).

**Figure 4:**
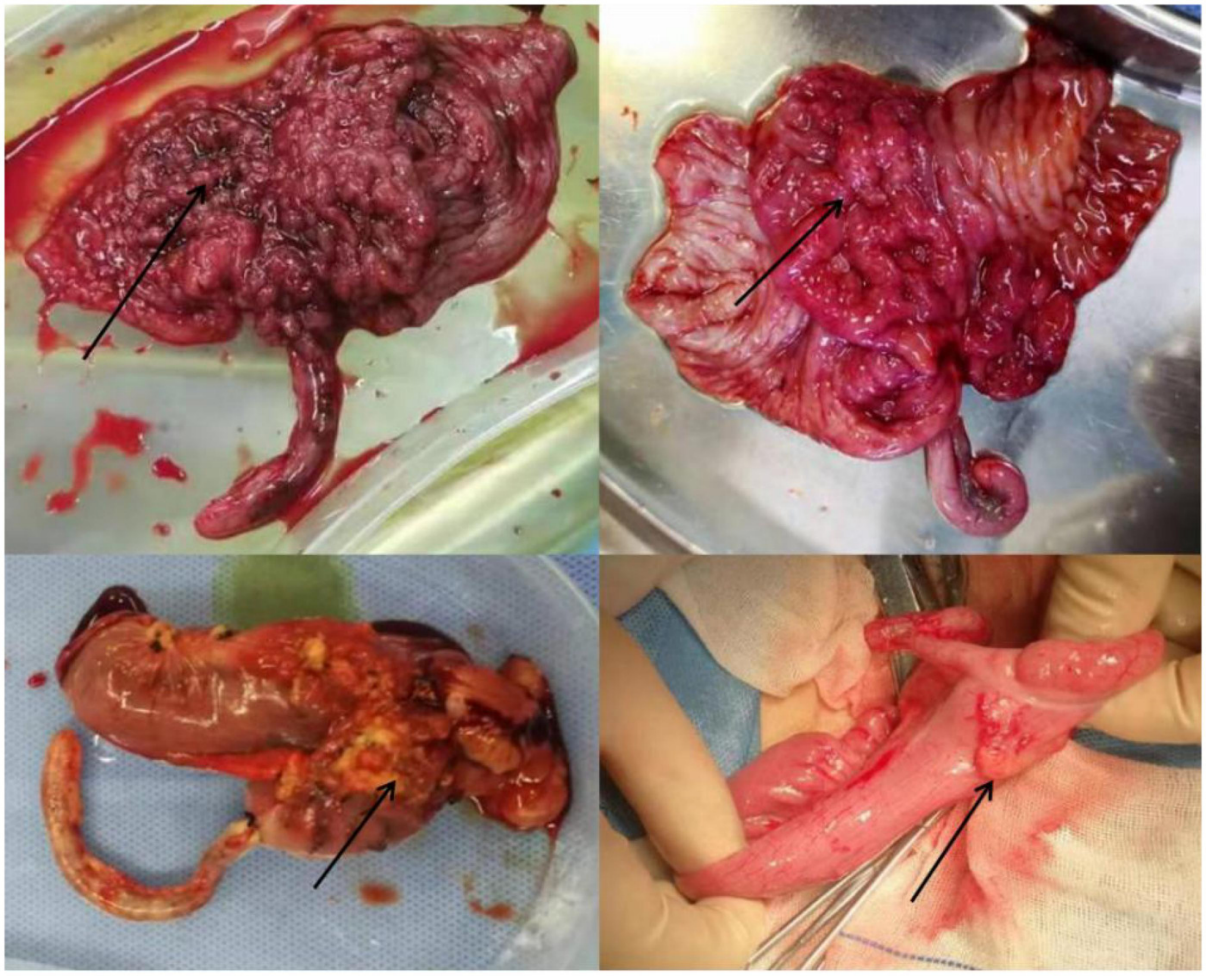
During the operation, lymphoid tissue hyperplasia was observed in the ileocecal region. Peyer's patches; mesenteric lymph nodes.

Based on intraoperative exploration, surgical methods are mainly divided into manual reduction of intussusception and resection and anastomosis of the diseased intestinal segment. Manual reduction is preferred through laparoscopic or open surgery for children with good intestinal vitality and no organic lesions. If the intestinal segment is necrotic, irreducible, or associated with a definite organic lesion (such as tumor, diverticulum), an intestinal resection and anastomosis (as shown in Figure 4) should be performed. Minimally invasive techniques have made significant progress, with laparoscopic surgery now an important option for treating children with intussusception, particularly in cases with a clear diagnosis and no severe complications. Compared with traditional open surgery, laparoscopic surgery has advantages such as less trauma, less postoperative pain, faster recovery, shorter hospital stay, and better aesthetics (82). For complex secondary intussusception or cases requiring intestinal resection, robot-assisted surgery can provide better operational accuracy (83). Indocyanine green near-infrared imaging and other fluorescence imaging techniques are helpful in real-time assessment of intestinal blood supply during operation, guiding precise resection range and preserving as much healthy intestinal tissue as possible (84). In children with large necrotic areas that cannot be anastomosed in one stage, a colostomy may be necessary, and a second-stage anastomosis can be performed after the condition stabilizes.

Overall, the choice of surgical method should be based on the specific condition of the child, the resources available at the medical institution, and the experience of the surgeon, aiming for minimal trauma, optimal prognosis, and individualized treatment.

### 5.3 Treatment for lymphoid tissue hyperplasia

For lymphoid tissue hyperplasia in the ileocecal region, a comprehensive treatment plan may include the following aspects: (1) Anti-inflammatory and immunomodulatory therapy: For lymphoid tissue hyperplasia caused by viral infection or inflammation, if the pathogen is identified, standardized anti-infective treatment can be considered. Some small-scale retrospective studies suggest that short-term use of glucocorticoids (such as dexamethasone) after the repositioning of intussusception may help reduce local edema and lower the risk of recurrence (77, 85). However, the application of glucocorticoids currently lacks high-level evidence and clear recommendations from authoritative guidelines, and its routine effectiveness and safety still need to be further verified through large-scale randomized controlled trials. (2) Intestinal microbiota intervention: Dysbiosis of the intestinal microbiota is considered one of the potential contributing factors of intussusception. Basic research has explored the possibility of regulating the intestinal microbiota by using probiotics (such as bifidobacteria and lactobacilli) or fecal microbiota transplantation (86, 87). However, these intervention measures are still in the preliminary research stage, lacking definite evidence from large-scale prospective clinical studies, and are not currently recommended as standard treatments. (3) Dietary management: Adjusting diet to avoid known allergens and limiting high-protein, high-fat foods may reduce excessive stimulation of the intestine, thereby decreasing abnormal immune responses. However, its specific role in preventing lymphoid tissue hyperplasia and recurrence of intussusception needs to be confirmed by further research. (4) Symptomatic supportive treatment: For children with clear infections (such as bacterial infections), antibiotics should be used based on results of drug sensitivity tests. For symptoms such as pain and vomiting, appropriate supportive treatment should be given.

## 6 Prognosis and follow-up

### 6.1 Prognosis evaluation

The overall prognosis of children with ileocecal lymphoid hyperplasia-related intussusception depends on the timing of presentation, pathological nature, treatment response, and individual baseline condition. Most children experiencing the first episode without intestinal necrosis recover well, with no impact on long-term quality of life after timely reduction; however, some groups still face the risks of recurrence, intestinal function damage, and even long-term growth and development limitations. (1) Course of disease and degree of intestinal injury: The time from onset to reduction is the most critical independent prognostic factor. Children who received intervention within ≤24 h of onset have a low incidence of intestinal necrosis and very few long-term complications; if the intervention exceeds 48 h, the risk of intestinal ischemia and perforation increases sharply, and even if the reduction is successful, there may still be complications such as intestinal stenosis and adhesive intestinal obstruction, and in severe cases, need for long-term parenteral nutrition support (79). (2) Nature of lymphoid hyperplasia: Benign reactive hyperplasia: It is often induced by infection or dietary stimulation, with a mild and localized degree of hyperplasia. After reduction, the lymphoid tissue can usually shrink spontaneously within 1–3 months due to the removal of the inducing factor. Recurrence risk is low, and no special long-term intervention is required. (3) Pathological hyperplasia or malignant lesion: If the pathology indicates a high proliferation index of Ki-67, positive EB virus-encoded RNA, or the presence of clonal gene rearrangement, it suggests a possibility of lymphoma or lymphoproliferative disease. Such children not only face the risk of intussusception itself but also need to bear additional risks such as the progression of the primary disease and adverse reactions to chemotherapy. In such cases, the 5-year overall survival rate is significantly lower than that in benign cases (88, 89). (4) Treatment response and complications: Children who successfully undergo the first enema reduction have the best prognosis. In cases of repeated recurrence, attention should be paid to organic lesions (such as Meckel's diverticulum, polyps), and repeated surgeries may increase the risks of short bowel syndrome and malnutrition. Children with intestinal perforation and peritonitis have a postoperative incidence of abdominal cavity infection and adhesive intestinal obstruction of 20%–30%, and some may require staged surgery, with significantly increased hospital stay and medical costs. (5) Individual baseline condition: Immune-deficient children have a recurrence rate 3–5 times higher than those without immune deficiency due to repeated antigen stimulation of lymphoid tissue. Premature infants and low-birth-weight infants have weak intestinal wall development, poor tolerance to ischemia, and a relatively worse prognosis.

### 6.2 Follow-up strategy

For children with intussusception caused by lymphoid hyperplasia in the ileocecal region, follow-up management should establish an individualized monitoring plan based on the risk of recurrence. In particular, risk assessment of the child should be conducted. For high-risk children with two or more recurrences or accompanying organic lesions, long-term or even lifelong follow-up is recommended. During the first year after reduction, abdominal ultrasound examinations should be conducted every 3–6 months to assess the regression of lymphoid tissue and intestinal peristalsis. Subsequently, annual reexaminations should be carried out until the child enters school age. For low-risk children, routine abdominal ultrasound examinations should be performed at 1, 3, and 6 months after reduction. The key to follow-up lies in multidimensional assessment. In terms of imaging, abdominal ultrasound is preferred, with a focus on monitoring the thickness of the ileocecal intestinal wall, the size of lymph nodes, and their blood flow signals. In terms of etiology, for children with recurrent episodes, whole-exome sequencing (WES) should be performed to rule out genetic immune deficiencies, or endoscopic examinations should be conducted to rule out organic lesions. In terms of functional aspects, regular assessment of the intestinal microbiota should be conducted, and probiotics should be appropriately supplemented to adjust intestinal function. In addition, attention should be paid to the risk of long-term intestinal adhesions. For children with chronic abdominal pain, low-dose CT screening can be performed. Families should maintain symptom diaries and implement dietary adjustments to aid early warning and intervention.

## 7 Discussion and outlook

The intestinal microbiota is a key regulatory factor in the pathogenesis of intussusception. Dysbiosis may disrupt the integrity of the intestinal barrier through the reduction of short-chain fatty acids (SCFA) and the excessive activation of Toll-like receptors. It is notable that viral infections (such as adenovirus) seem to exacerbate dysbiosis by damaging the mucosal barrier, creating a “virus–bacteria synergy” that amplifies lymphoid tissue hyperplasia. Future research should prioritize longitudinal cohort studies to verify the causal relationship and explore intervention measures targeting the microbiota (such as precise probiotics or fecal microbiota transplantation) as adjunct therapies to reduce recurrence.

In recent years, significant progress has been made in understanding the role of lymphoid tissue hyperplasia in the ileocecal region in the mechanism of intussusception. The lymphoid tissue within the intestinal wall mainly consists of PPs and lymphoid follicles, which are widely distributed in the mucosal and submucosal layers of the ileum and cecum. In children, especially infants, the development of these intestinal lymphoid tissues is particularly remarkable. Their volume is significantly larger than that of adult lymphoid tissues and becomes an important component of the intestinal immune defense during the childhood stage. There are a large number of lymph nodes in the mesentery of the ileocecal region, and these lymph nodes are interconnected with the lymphoid tissue within the intestinal wall, jointly forming a local immune network. Studies have shown that lymphoid tissue hyperplasia caused by viral infections in the mesenteric lymph nodes and in the ileocecal region is an important factor in inducing intussusception. When affected by viral infections (such as adenovirus, rotavirus) or inflammatory stimuli, the lymphoid tissue in the ileocecal region will become congested, edematous, and proliferative, resulting in thickening of the intestinal wall and the formation of local “polypoid protrusions”. Combined with the high activity of the ileal wall in the ileocecal region and the hypertrophy of the ileocecal valve and other anatomical features, the hyperplastic lymphoid tissue may become the “entry point” for intussusception, causing the intestinal wall to be pulled during intestinal peristalsis rhythm disorder, resulting in intussusception. In surgical cases, the lymph nodes in the ileocecal region can also show varying degrees of enlargement, and in some cases of intussusception, the aggregated lymph nodes in the ileal wall display signs of local hypertrophy, edema, and depression, further confirming the central role of lymphoid tissue in the pathogenesis. Advances in imaging techniques have significantly improved the diagnostic accuracy of intussusception. Ultrasound examination can provide clear visualization of intussusception and enlarged mesenteric lymph nodes, supporting early diagnosis. CT enhanced scanning is of great significance in evaluating whether there is ischemia or necrosis in the intestinal tube. Air enema and water pressure enema under the guidance of B-ultrasound can not only be used for diagnosis but also for treatment, enabling most children to avoid surgery. For patients with recurrent intussusception, if no significant changes in abdominal lymph nodes or intestinal malformations are found by color Doppler ultrasound or other examinations, intestinal endoscopy can clearly determine the status of lymphoid tissue hyperplasia in the intestine and determine the location of the lesion. The development of minimally invasive surgical techniques provides new options for the treatment of intussusception. Through transumbilical single-incision laparoscopic-assisted resection of the ileocecal lesion caused by intussusception, good results have been achieved, demonstrating the advantages of minimally invasive surgical techniques in the treatment of intussusception in children and laying the foundation for individualized treatment.

Future research should focus on the following aspects: (1) deep exploration of the molecular mechanism of ileocecal lymphoid tissue hyperplasia to identify key regulatory factors; (2) development of more precise recurrence prediction models, incorporating more risk factors; (3) using spatial multi-omics technology to analyze the interaction map of cells in the lymphoid tissue microenvironment; (4) designing targeted treatment plans for lymphoid tissue hyperplasia; (5) development of nanorobots to achieve *in situ* repair in the intestine; and (6) conducting large-scale sample studies in multiple centers to verify the general applicability of existing research results.

## 8 Conclusion

Lymphoid tissue hyperplasia in the ileocecal region of children is an important pathological factor in the development of intussusception. Its occurrence involves multiple dimensions of interactions, including the anatomical structure, immune environment, microbial community, and mechanical force transmission. Viral infection, inflammatory response, microecological imbalance, and genetic susceptibility jointly promote the pathological hyperplasia of lymphoid tissue, thereby forming the mechanical fulcrum of intussusception. Imaging modalities such as ultrasound, CT, and MRI multimodal fusion are key diagnostic methods, and the application of artificial intelligence has significantly improved the efficiency and accuracy of diagnosis. A stepwise comprehensive treatment plan, including enema reduction, drug regulation, fecal microbiota transplantation, and minimally invasive surgery, embodies the concept of personalized precision medicine, providing new options for refractory cases in terms of immune regulation and microecological intervention. In the future, it is necessary to strengthen translational research between basic and clinical fields, using cutting-edge technologies such as organoids, single-cell sequencing, and gene editing, to deeply explore molecular mechanisms, develop targeted nanomedicines and microbial therapies, establish a precise diagnosis and treatment system based on multi-omics integration, and ultimately achieve the comprehensive goals of predicting, preventing, and precisely treating intussusception.
